# Exploring the effects of long-term physical exercise on persistent and inhibitory self-control: quasi-experimental research

**DOI:** 10.3389/fspor.2025.1543481

**Published:** 2025-03-18

**Authors:** Linjing Cheng, Huahui Qin, Yin Yang, Junhua Dang

**Affiliations:** ^1^ School of Business Foreign Languages, Shenzhen Polytechnic University, Shenzhen, China; ^2^ School of Psychology, Beijing Sport University, Beijing, China; ^3^ School of Humanities and Social Sciences, Xi’an Jiaotong University, Xi’an, China; ^4^ Department of Surgical Sciences, Uppsala University, Uppsala, Sweden

**Keywords:** physical exercise, strength model, persistent self-control, inhibitory self-control, self-control

## Abstract

This study aims to explore the effects of long-term physical exercise on different types of self-control, with a focus on persistent and inhibitory self-control. Two experiments were conducted using dual-task paradigms. In Experiment 1, the E-crossing task served as the depletion task, and the grip task was used to measure persistent self-control. Results indicated that long-term exercisers exhibited significantly better persistent self-control than non-exercisers, *F*(1, 54) = 6.55, *p* = .013, η*_p_^2^* = 0.11. Experiment 2 employed the Stroop task as the detection task to measure inhibitory self-control. No significant differences were found between the exercise and non-exercise groups in inhibitory self-control performance. These findings suggest that long-term physical exercise may enhance persistent self-control, but its effects on inhibitory self-control remain unclear. The study addresses potential confounding factors, such as task-specific effects and baseline performance differences, and highlights the need for future research to explore diverse self-control tasks and establish causal relationships. The results contribute to the understanding of self-control training and provide insights into the domain-specific effects of physical exercise on self-control.

## Introduction

Physical exercise refers to planned, organized, and repeated physical activity with specific goals aimed at maintaining vitality and improving fitness (1). A substantial body of research consistently demonstrates the benefits of regular physical exercise, including improvements in mental health (2), as well as enhanced memory, learning ability (3), and self-control (4–8). Recent studies have also shown that physical exercise promotes neuroplasticity by increasing brain-derived neurotrophic factor (BDNF) (9), providing a physiological basis for the benefits of physical exercise.

The strength model of self-control, proposed by Baumeister et al. (10), is a widely accepted framework for understanding self-control. According to this model, self-control relies on a finite resource that becomes temporarily depleted with continuous use, leading to a state of ego depletion in which individuals struggle to sustain self-control behaviors and often fail in self-control tasks. Furthermore, this model posits that self-control resources are general, meaning depletion in one domain can impair performance across other self-control domains. Baumeister et al. (10) provided evidence for this hypothesis using a dual-task paradigm and introduced the training hypothesis, which proposes that self-control, like a muscle, can be strengthened through consistent practice, thereby reducing the effects of ego depletion over time (6, 11–13). This theory holds significant implications in sports science, offering a complementary perspective on the psychological mechanisms underlying the effects of exercise on self-control. Long-term physical activity may mitigate ego depletion by expanding psychological resources or accelerating resource recovery (6), ultimately enhancing performance across various domains of self-control.

Self-control in sports is often categorized into persistent self-control and inhibitory self-control (14, 15). Persistent self-control involves sustained effort in challenging situations without altering the level or direction of effort, a capacity commonly observed in endurance sports such as running and swimming. Tasks that measure persistent self-control include handgrip tasks (16), pain tolerance tasks (5, 8), and wall-sit tasks (31). In contrast, inhibitory self-control focuses on the conscious suppression of automatic or habitual responses, often required in reactive sports such as soccer or badminton. Common tasks used to measure inhibitory self-control include the Stroop task (17, 18), the Go/no-go task (31), and thought suppression tasks (19).

Preliminary evidence suggests that physical exercise positively influences both persistent and inhibitory self-control. For example, Jones et al. (5) found significant improvements in ischemic pain tolerance following 6 weeks of physical exercise. Similarly, Zou et al. (8) reported that participants who engaged in 5 weeks of physical exercise maintained their pain tolerance over time, while a control group exhibited declines. These findings suggest that physical exercise enhances persistent self-control. Evidence for the effects of exercise on inhibitory self-control includes improvements on tasks like thought suppression (6) and the Stroop task following aerobic exercise interventions (7, 20). Collectively, these findings indicate that physical exercise may improve both forms of self-control.

Despite these promising findings, the effects of physical exercise on self-control remain a topic of debate. Some researchers argue that self-control is a core component of executive functioning (17, 18), and many studies have used executive function as a proxy for self-control [e.g., (4, 18, 20)]. However, self-control is a multidimensional construct that is not strongly correlated with executive function (21), making it problematic to generalize findings about executive function to self-control. Additionally, much of the existing research has focused on specific types of exercise (e.g., karate training; Wen et al., 2020), which limits the generalizability of the results. Furthermore, many studies have been conducted in laboratory settings, where biases such as expectancy effects (22) and participation for monetary compensation (23) may influence outcomes. Laboratory interventions are often short-term, typically lasting around 2 months or less, and few studies have investigated the effects of long-term exercise on self-control.

To address the limitations of existing research, this study examines the effects of physical exercise on persistent and inhibitory self-control within the framework of the self-control strength model. First, the study employs a dual-task paradigm based on the self-control strength model. By comparing self-control performance between an exercise group and a non-exercise group following task depletion, this approach highlights the concept of self-control resources and clarifies the distinction between self-control and executive functions. Second, the self-control strength model posits that self-control improves through sustained training. To explore this, the study investigates the long-term effects of physical exercise on self-control abilities by comparing the performance of regular exercisers and non-exercisers on self-control tasks. According to the model's general resource hypothesis, depletion of self-control resources affects performance across all self-control domains. If physical exercise enhances self-control resources, then long-term exercisers should outperform non-exercisers on a variety of self-control tasks. Thus, this study examines the impact of physical exercise on both persistent and inhibitory self-control. Participants will be divided into two groups: an exercise group consisting of individuals who have engaged in regular physical activity for at least 6 months, and a non-exercise group comprising individuals who have not participated in any exercise. No restrictions will be placed on the type of exercise to ensure ecological validity. The research consists of two experiments. The first experiment utilizes the E-crossing task as a depletion task and the handgrip task as a probe task to assess the effects of physical exercise on persistent self-control. The second experiment employs the E-crossing task and the Stroop task to evaluate inhibitory self-control. Based on previous research, this study hypothesizes that individuals who engage in long-term, regular physical exercise will demonstrate superior performance on both persistent and inhibitory self-control tasks compared to non-exercisers.

## 2 Experiment 1: effect of exercise status on persistent self-control

To investigate differences between exercisers and non-exercisers on a self-control task following cognitive depletion, this study employed a 2 by 2 mixed quasi-experimental design. Exercise status (exercisers vs. non-exercisers) served as the between-participant variable, while timepoint (pre-test vs. post-test) was the within-participant variable. The dependent variable was the duration participants sustained their grip on the handle during the grip task, which served as a measure of persistent self-control.

### 2.1 Methods and materials

#### 2.1.1 Participants

Fifty-eight healthy college students participated in this study. Participants were grouped based on definitions provided by the *Physical Exercise Guidelines Advisory Committee* (1). Ethical approval for the study was granted by the Institutional Review Board of Beijing Sport University. This study adopted the criteria issued by the State General Administration of Sport of China: physical exercise must occur at least three times per week, with each session lasting 30 min or more at a moderate or higher intensity. The screening criteria for the exercise group were as follows: participants must have engaged in purposeful and planned exercise for at least 6 months, with a frequency of three or more sessions per week, each lasting at least 30 min at a moderate or higher intensity. For the non-exercise group, the criteria were: no purposeful exercise or structured program in the past 6 months, no more than two exercise sessions per month, no sessions exceeding 30 min, and an intensity level below moderate. According to the central limit theorem, when the sample size of each experimental group reaches 30, the sample distribution tends to approximate normality, even if the population distribution is not perfectly normal. However, due to the limited number of eligible participants in the exercise group and budgetary constraints, only 27 participants were ultimately included (20 females, mean age = 21.44 ± 2.08 years). The non-exercise group included 30 participants (26 females, mean age = 21.17 ± 1.80 years). A chi-square test revealed no significant difference in gender distribution between the groups (*χ*² = 0.28, *p* = .594). An independent samples *t*-test indicated no significant difference in age between the groups, *t*(55) = 0.29, *p* = .591.

#### 2.1.2 Material

*The grip task* required participants to use an R-type Li-Ning gripper with a squeeze force of 10 kg. This gripper, measuring 15 cm × 11 cm and weighing approximately 122 g, consisted of a handle connected by a metal spring. Participants were instructed to squeeze the handles together forcefully enough to clamp a piece of A4 paper inserted between them. A minimum force of 10 kg was necessary to hold the paper securely. If the participant's force diminished and the handles began to release, the paper would fall, signaling the end of the task. The experimenter started a timer when the paper was clamped and stopped it when the paper fell. To eliminate performance biases, participants were not provided with feedback during the task and were not allowed to view time-keeping devices. Previous research indicates that maintaining grip strength in this context is primarily a measure of self-control, with minimal influence from individual muscle strength (16, 24). Therefore, the grip task is a valid tool for assessing self-control.

*The E-crossing task*, serving as the cognitive depletion task, required participants to suppress the automatic habit of copying words as written and instead apply specific rules. Fifty-seven words were selected from the IELTS Disorganized Thesaurus and presented in a Word document. Initially, participants copied the 57 words, omitting all instances of the letter “e.” After completing this task, a new rule was introduced: participants had to omit “e” unless the second letter to the left or right of the “e” was a vowel. For example, in the word “department,” the “e” is retained because the second letter to its right is “a,” a vowel. The entire task took approximately 10 min to complete. Prior studies have demonstrated that performing the E-crossing task under these conditions significantly depletes cognitive resources (15, 25).

*Subjective depletion* was assessed using a 7-point rating scale. Participants rated the difficulty of the task, the effort they exerted, and their level of self-control during the E-crossing task. Higher ratings on these dimensions indicated greater cognitive depletion from the task (26).

#### 2.1.3 Procedure

This study strictly followed ethical guidelines for psychological research. An ethical application was submitted to and approved by the relevant authorities to ensure the protection of participants’ rights and interests. This experiment was conducted in the winter of 2021. Since WeChat is widely used among university students, and the sharing and forwarding through Moments can effectively expand the reach of the recruitment, this study recruited participants through a combination of WeChat Moments and campus announcements, providing detailed information on the screening criteria. The principal examiner verified participants' eligibility by confirming their basic information and exercise habits before scheduling laboratory appointments. Upon arrival at the lab, participants received a comprehensive introduction to the study. This briefing included the purpose of the research, the experimental procedures, potential risks or discomforts, and their right to withdraw from the experiment at any time without penalty. Participation was confirmed as entirely voluntary, and participants were then asked to sign an informed consent form. The experimental procedure began with a pre-test of the grip strength task. Following this, participants took a 5-min break. After the break, they completed the depleting task (the E-crossing task) and the measure of subjective depletion. Finally, participants performed a post-test of the grip strength task. Both the exercise and non-exercise groups followed the same experimental procedure.

#### 2.1.4 Statistical analysis

Statistical analyses were conducted using SPSS 21.0. Extreme values were identified using a 3-standard-deviation criterion, leading to the exclusion of one participant whose pre-test grip duration (330.045 s) and post-test grip duration (306.083 s) significantly deviated from the group norms. As a result, the final dataset included 26 participants in the exercise group and 30 participants in the non-exercise group. The difference in subjective depletion was assessed using independent samples *t*-tests. Differences in persistent self-control between exercisers and non-exercisers were analyzed through repeated-measures ANOVA, supplemented by simple effects tests to further explore specific group differences.

### 2.2 Results

#### 2.2.1 Subjective depletion

Descriptive statistics are presented in Table 1. Exercise status was the independent variable, and independent samples *t*-tests were conducted with self-reported difficulty, effort, and self-control as the dependent variables. The differences between the exercise and non-exercise groups on all dimensions were not significant: difficulty, *t* (54) = 0.98, *p* = .334; effort, *t* (54) = 0.02, *p* = .981; self-control *t* (54) = 1.01, *p* = .319. These results suggest that exercisers and non-exercisers were similarly engaged in the E-crossing task, and any observed behavioral differences are unlikely to be attributed to variations in initial task engagement.

**Table 1:** Descriptive statistics of experiment 1.

Dependent variables	Exercise group M ± SD	Non-exercise group M ± SD
Difficulty	3.58 ± 1.24	3.27 ± 1.14
Effort	3.81 ± 1.13	3.80 ± 1.21
Self-control	4.54 ± 1.39	4.17 ± 1.37
Grip task pre-test	111.52 ± 58.27	100.13 ± 55.01
Grip task post-test	105.20 ± 54.41	74.14 ± 35.61

#### 2.2.2 Analysis of grip task

**Figure 1:**
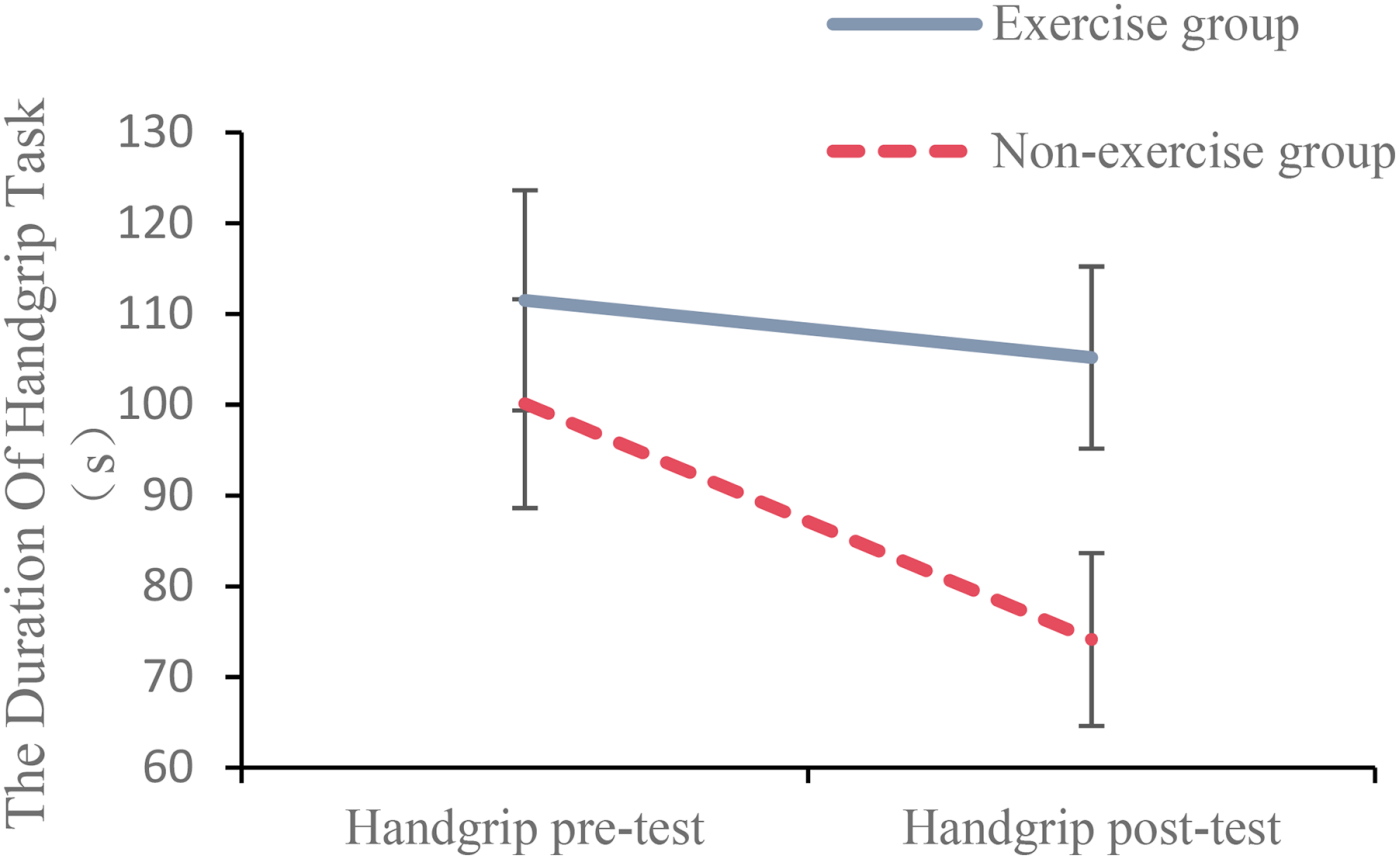
Interaction test plot of grip handle endurance duration.

Descriptive statistics are shown in Table 1.The results of the repeated measures ANOVA showed a non-significant main effect of exercise status, *F*(1, 54) = 2.73, *p* = .104, ƞ*_p_^2^* = 0.05, a significant main effect of time, *F*(1, 54) = 11.14, *p* = .002, ƞ*_p_^2^* = 0.17, and a significant interaction between time and exercise status *F*(1, 54) = 4.13, *p* = .047, ƞ*_p_^2^* = 0.07. Simple effects results found (see Figure 1) that there was no significant difference between the two groups of participants in terms of the length of time they persisted in the handgrip pre-test task, *F*(1, 54) = 0.57, *p* = .455, ƞ*_p_^2^* = 0.01. The exercise group persisted significantly longer than the non-exercise group in the handgrip posttest task, *F*(1, 54) = 6.55, *p* = .013, ƞ*_p_^2^* = 0.11.

## 3 Experiment 2: effect of exercise status on inhibitory self-control

To investigate differences in inhibitory self-control between adherent physical exercisers and non-exercisers under a state of depletion, the study employed a between-subject design. The independent variable was exercise status (exercisers vs. non-exercisers), and the dependent variable was performance on the Stroop task.

### 3.1 Methods and materials

#### 3.1.1 Participants

Ethical approval for the study was granted by the Institutional Review Board of Beijing Sport University. The study included 60 healthy college students, and the grouping method was consistent with Experiment 1. According to the central limit theorem, when the sample size of each experimental group reaches 30, the sample distribution tends to approximate a normal distribution, even if the population distribution is not perfectly normal. However, due to the limited number of participants meeting the criteria for the exercise group, as well as budgetary constraints, only 29 participants were ultimately included. The study comprised 29 participants in the exercise group (17 females, mean age = 20.28 ± 1.98 years) and 31 participants in the non-exercise group (28 females, mean age = 21.03 ± 6.02 years). A chi-square test revealed a significant difference in gender distribution between the exercise and non-exercise groups, *χ*² = 8.03, *p* = .005. However, an independent sample *t*-test showed no significant difference in age between the two groups: *t* (58) = −0.64, *p* = .522. Given the significant gender difference, gender was included as a covariate in subsequent statistical analyses.

#### 3.1.2 Material

**Figure 2:**
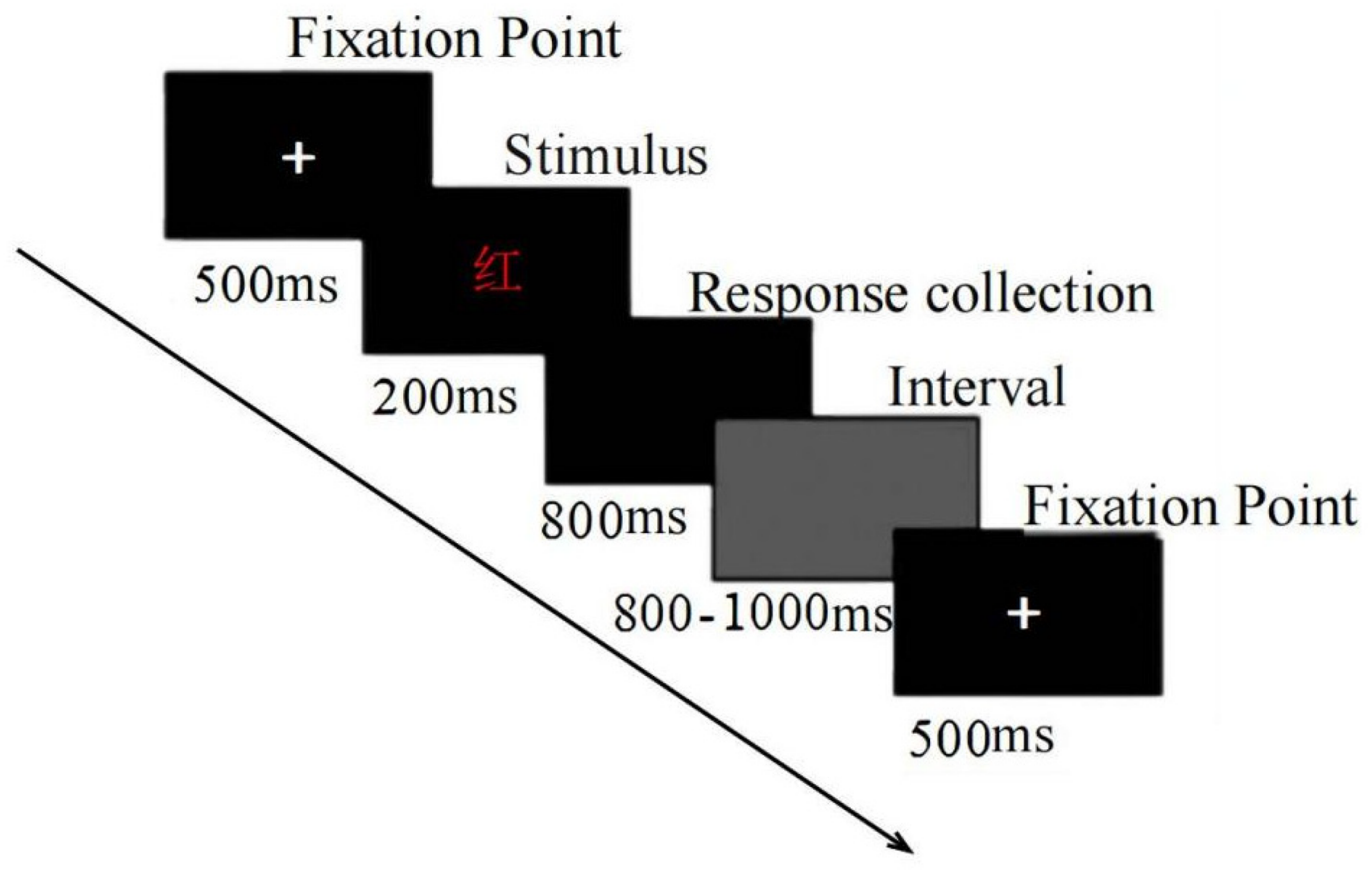
Stroop task flow diagram.

The E-crossing task and the measure of subjective depletion were the same as in Experiment 1. The Stroop task, a common measure of inhibitory self-control, consisted of both color-word congruent and color-word incongruent trials. In this task, participants were instructed to ignore the meaning of the words and respond based on the color in which the word was presented. The participants' self-control was assessed based on their response times and accuracy. In this study, four Chinese color words—red, green, yellow, and blue—were used across a total of 192 trials, following the procedure outlined in previous research [e.g., Zhang & Zhang (13)]. There were 84 congruent trials, where the color of the word matched its meaning (e.g., “RED” displayed in red), and 108 incongruent trials, where the color of the word did not match its meaning (e.g., “RED” displayed in blue). Participants were instructed to vocalize the meaning of the word after seeing the stimulus. The specific process of the task is shown in Figure 2.

#### 3.1.3 Procedure

This experiment was conducted in the winter of 2021. After signing the Informed Consent Form, participants first completed a practice session for the Stroop task, consisting of 16 trials. At the end of the practice, the experimenter asked participants whether they understood the rules, were familiar with the correspondence between the keys and the colors, and if they felt ready to proceed. If any participant indicated they were unsure, the practice session was repeated. Once the participant confirmed readiness, the practice session was concluded, and a 5-min break was scheduled. After the break, participants performed the depletion task (the E-crossing task), followed by completing the measure of subjective depletion. Finally, they performed the Stroop task. The experimental procedure was identical for both the exercise and non-exercise groups.

#### 3.1.4 Statistical analysis

Statistical analyses were conducted using SPSS 21.0. Extreme values were identified using a 3-standard-deviation criterion, resulting in the exclusion of two extreme values (inconsistent correct rates of 69.4% and 71.2%). Consequently, 28 participants in the exercise group and 30 participants in the non-exercise group were included in the statistical analysis. Independent samples *t*-tests were used to examine subjective depletion. To test for differences in inhibitory self-control between exercisers and non-exercisers, analysis of covariance (ANCOVA) was conducted, with exercise status as the independent variable, gender as the covariate, and performance on the Stroop task as the dependent variable. The Stroop effect was calculated as the difference between the congruent and incongruent trials, and this was measured using both response time (RT) and accuracy rate as separate indicators.

### 3.2 Results

#### 3.2.1 Subjective depletion

Descriptive statistics are shown in Table 2. The exercise and non-exercise groups did not show a significant difference in difficulty, *t* (56) = 1.23, *p* = .224; effort, *t* (56) = 0.98, *p* = .332; self-control *t* (56) = 1.28, *p* = .204. These results suggest that exercisers and non-exercisers were similarly engaged in the E-crossing task, and any observed behavioral differences are unlikely to be attributed to variations in initial task engagement.

**Table 2:** Descriptive statistics of experiment 2.

Dependent variables	Exercise group M ± SD	Non-exercise group M ± SD
Difficulty	3.79 ± 1.23	3.40 ± 1.16
Effort	4.00 ± 1.15	3.70 ± 1.18
Self-control	4.57 ± 0.84	4.23 ± 1.14
Stroop effect RT	83.79 ± 48.59	85.05 ± 37.71
Stroop effect accuracy	0.02 ± 0.03	0.02 ± 0.03

#### 3.2.2 Stroop task performance

Descriptive statistics are shown in Table 2. An ANCOVA with gender as a covariate showed that the exercise and non-exercise groups did not differ in either the Stroop effect calculated from RT, *F*(1, 57) = 0.23, *p* = .635, or the Stroop effect calculated from accuracy, *F*(1, 57) = 0.02, *p* = .896.

## 4 Discussion

This paper examined the effects of physical exercise on self-control using two studies that presented inconsistent results. Long-term exercisers performed significantly better on a persistent self-control task than the non-exercise group. However, no differences were found between the exercise and non-exercise groups on an inhibitory self-control task.

### 4.1 The effect of physical exercise on self-control

In Experiment 1, we used a dual-task paradigm, with the E-crossing task as the depletion task and the grip strength task as the detection task, to examine the effects of exercise on persistent self-control. The results indicated that long-term exercisers demonstrated better persistent self-control than non-exercisers, which is consistent with the findings of Jones et al. (5) and Zou et al. (8). For example, Jones et al. (5) showed that a 6-week aerobic exercise intervention significantly increased participants' ischemic pain tolerance, while Zou et al. (8) found that a 5-week exercise training program maintained the stability of pain tolerance. These studies suggest that physical exercise may improve persistent self-control by enhancing physiological endurance or the ability to recover resources. However, given that both physical exercise and the grip task involve physical exertion, one might argue that physical exercise has a higher transfer potential for tasks based on muscular activity. This perspective could explain why long-term exercisers outperformed non-exercisers on the grip task.

In response to this viewpoint, two counterarguments are presented. First, previous research has shown that handgrip endurance duration is not significantly related to an individual's physical strength. The time spent maintaining handgrip is unaffected by exertion intensity (24) and is not correlated with maximum grip strength (27). Second, in the pretest of Experiment 1, no significant differences were observed between the exercise and non-exercise groups in the handgrip test, suggesting that physical exercise does not directly influence baseline performance in this task. Taken together, these findings indicate that the superior performance of the exercise group in the persistent self-control task cannot be solely attributed to overlapping muscle activity. To further validate this hypothesis, future research should incorporate alternative task types, such as computer-based tasks unrelated to physical strength—like maze tasks—as dependent variables. This approach would help deepen our understanding of the relationship between physical exercise and self-control while yielding more stable and generalizable results.

In Experiment 2, the detection task was changed to the Stroop task to explore the effects of exercise on inhibitory self-control. Results showed no significant differences between the exercise and non-exercise groups in inhibitory self-control performance. This finding is inconsistent with previous studies (4, 7, 18). For example, Smiley-Oyen et al. (7) observed a significant improvement in Stroop task performance following acute aerobic exercise intervention, and Baker et al. (4), in a randomized experimental and control group study, found that 6 months of exercise could improve performance on inhibitory self-control tasks such as the Stroop task in elderly women. The discrepancy may be due to differences in experimental paradigms. While most prior studies directly measured inhibitory self-control after physical exercise interventions, this study employed a dual-task paradigm to assess inhibitory self-control in long-term exercisers following depletion. The differences in measurement methods and experimental structure may explain the inconsistent results. While most prior studies directly measured inhibitory self-control after physical exercise interventions, this study employed a dual-task paradigm to assess inhibitory self-control in long-term exercisers following depletion. The differences in measurement methods and experimental structure may be one of the reasons for the inconsistent results.

This negative result may be related to task-specific factors. For example, the Stroop task, as an inhibitory self-control task, may not be sensitive enough to capture subtle differences between long-term exercisers and non-exercisers. Another possibility is that both groups had relatively high baseline levels of inhibitory control, and their self-control resources were sufficient, leading to a ceiling effect that made it difficult to observe differences between the two groups in the Stroop task.

Additionally, these inconsistent results may reflect subtle differences within the theoretical framework of self-control. Similar findings have been reported in previous studies. For example, Muraven et al. (12) found that diet and posture control training improved performance on the depletion-based grip task, while emotional control training did not show this effect. Oaten and Cheng (6) discovered that self-control training improved performance on a visual tracking task, but did not lead to significant changes in sleep or tooth-brushing habits. Hui et al. (11) observed that participants receiving self-control training had longer cold tolerance times, but lower tooth-brushing frequency than the control group. These results suggest that not all types of self-control training universally enhance self-control. Moreover, debates persist about whether self-control can be enhanced through training, with both supporting (22) and opposing (28) evidence. The results of this study align with the notion that single-domain self-control training may not universally enhance self-control across all domains.

To address these uncertainties, future research could include more tasks to further verify these findings and avoid task-specific effects. Additionally, more tasks directly assessing inhibitory control could be introduced, and physiological changes in participants could be observed using more advanced instruments like EEG (electroencephalography) and TMS (transcranial magnetic stimulation). These tools, along with more comprehensive and precise measures, would allow for a better evaluation of the impact of exercise on inhibitory self-control. Finally, reaction inhibition tasks could be measured under varying depletion levels to obtain more discriminative results.

In conclusion, although this study provides valuable insights into the effects of long-term exercise on self-control, a more diverse set of tasks and methods should be employed in future research to fully understand the relationship between physical exercise and the various dimensions of self-regulation.

### 4.2 Innovations and contributions

Building on the concept of physical exercise and the definition of a regular exercise population, this study takes an innovative approach by selecting an exercise-based population framework, enriching research on the relationship between long-term exercise and self-control. Grounded in the self-control strength model, the study examines the effects of physical exercise on different types of self-control, using the ability to sustain self-control tasks after exertion as an indicator. This approach effectively distinguishes self-control from executive function, ensuring a more precise focus on self-control mechanisms. Unlike previous studies that examined the effects of physical exercise on only one type of self-control or a single task (4, 7) which, despite their use of standardized tasks, may still raise concerns about task specificity—this study adopts a widely recognized classification of self-control. It selects tasks representing persistence and inhibitory self-control (the grip task and the Stroop task), thereby reducing task-specific effects and providing a clearer understanding of the relationship between physical exercise and self-control.

Although the sample consisted of Chinese university students, the findings may extend to broader populations. The self-control enhancement mechanism has been validated across age and cultural groups [e.g., children, older adults (7, 29)]. Furthermore, exercise-induced neuroplasticity, a universal pathway for self-control improvement (4, 9), supports the generalizability of these findings. While cultural and age-related moderators warrant careful consideration, existing evidence aligns with broader extrapolation.

In practical terms, this study not only encourages individual participation in physical exercise but also offers new insights for those seeking to enhance their self-control. Additionally, it reveals the specific effects of physical exercise on different types of self-control, particularly highlighting its differential impact on persistence and inhibitory self-control. This distinction provides refined guidance for improving self-control, suggesting that exercise regimens should be tailored to address specific self-control needs.

Future research should explore the differential effects of exercise modalities (e.g., intensity, frequency) on various dimensions of self-control and validate these findings in diverse populations (e.g., adolescents, clinical groups) to refine personalized interventions. In conclusion, this study offers preliminary theoretical support for designing individualized training programs, with significant practical and applied value.

### 4.3 Limitations

First, most existing studies on self-control training use a single indicator to represent the effectiveness of self-control training (26, 30). This study also selected only one task indicator for each type of self-control; future studies should incorporate more tasks to further validate the results. Second, this study did not adopt an experimental design, but instead explored the differences between two groups (exercise group and non-exercise group) in self-control tasks. Although the study provides preliminary evidence for the relationship between physical exercise and self-control, causal relationships cannot be determined. Future intervention studies (such as randomized controlled trials) are crucial for verifying the causal effects of physical exercise on self-control. Third, this study did not restrict the specific types of exercise (such as intensity and frequency), which may introduce confounding variables. Future research should further explore the mechanisms by which different exercise modes affect self-control. Additionally, resource limitations (such as lack of funding support) have impacted the rigor of the experimental design and the representativeness of the sample. For example, this study was unable to recruit a larger, more diverse group, nor did it use more precise measurement tools [such as transcranial magnetic stimulation (TMS), EEG, etc.] to further explore the impact of physical exercise on self-control. Future research should expand the sample size and diversity, and adopt more precise measurement tools to confirm their broader applicability and deepen our understanding of the related field. Furthermore, the choice of experimental tasks (such as grip strength and Stroop tasks) may have task-specific effects on the results. Therefore, future research should combine multidimensional indicators and stricter control conditions to improve the generalizability and reliability of the conclusions. Finally, although there is no conflict of interest in the study, and the experimenters did not disclose the experimental intent, self-report data from participants may still be subject to social desirability bias.

## Data Availability

The datasets presented in this study can be found in online repositories. The names of the repository/repositories and accession number(s) can be found below: https://osf.io/a92e6/?view_only=547e129afa4a4aa285283d5857c830b5.
